# *De novo* Whole‐Genome Assembly of the 10‐Gigabase *Fokienia Hodginsii* Genome to Reveal Differential Epigenetic Events Between Callus and Xylem

**DOI:** 10.1002/advs.202402644

**Published:** 2024-09-04

**Authors:** Jundong Rong, Yushan Zheng, Zeyu Zhang, Jun Zhang, Yuying Gu, Tian Hua, Mengna Zhao, Lili Fan, Zhiwen Deng, Yanmei Pan, Bingjun Li, Liguang Chen, Tianyou He, Lingyan Chen, Jing Ye, Hangxiao Zhang, Lianfeng Gu

**Affiliations:** ^1^ College of Forestry Fujian Agriculture and Forestry University Fuzhou 350002 China; ^2^ Fujian Provincial Key Laboratory of Haixia Applied Plant Systems Biology School of Future Technology Fujian Agriculture and Forestry University Fuzhou 350002 China; ^3^ College of Landscape Architecture Fujian Agriculture and Forestry University Fuzhou 350002 China

**Keywords:** alternative splicing, direct RNA sequencing, Fokienia hodginsii, poly(A) length, stem‐differentiating xylem

## Abstract

*Fokienia hodginsii* (*F*. *hodginsii*), belonging to the genus Fokienia of the Cupressaceae. *F*. *hodginsii* has significant application value due to its wood properties and great research value in evolutionary studies as a gymnosperm. However, the genome of *F*. *hodginsii* remains unknown due to the large size of gymnosperms genome. Pacific Bioscience sequencing, Hi‐C mapping, whole‐genome Bisulfite Sequencing (BS‐Seq), long‐read isoform sequencing (Iso‐Seq), direct RNA sequencing (DRS), quantitative proteomics, and metabonomics analysis are employed to facilitate genome assembly, gene annotation, and investigation into epigenetic mechanisms. In this study, the 10G *F*. *hodginsii* genome is assembled into 11 chromosomes. Furthermore, 50 521 protein‐coding genes are annotated and determined that 65% of *F*. *hodginsii* genome comprises repetitive sequences. It is discovered that transposable element (TE)‐including introns is associated with higher expression. The DNA methylome of *F*. *hodginsii* reveals that xylem has a higher DNA methylation level compared to callus. Moreover, DRS reveals the significant alterations in RNA full‐length ratio, which potentially associated with poly(A) length (PAL) and alternative polyadenylation (APA). Finally, the morphology measurement and metabonomics analysis revealed the difference of 14 cultivars. In summary, the genomes and epigenetics datasets provide a molecular basis for callus formation in the gymnosperm family.

## Graphical abstract

This study presents a chromosome‐level genome assembly of *Fokienia hodginsii*, a gymnosperm with valuable wood properties. Utilizing advanced sequencing technologies, the research constructs a 10G genome across 11 chromosomes, annotates over 50 000 genes. Key findings include the impact of transposable elements on gene expression and distinct DNA methylation patterns in different tissues, providing insights into callus formation.

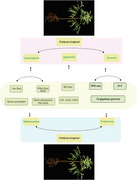

## 1 Introduction

*F*. *hodginsii* is a montane evergreen forest species, which exhibits great genetic diversity^[^ ^1^ ^]^ and has a widespread distribution in North Vietnam, and extends to southeastern China.^[^ ^2^ ^]^ The growth of *F*. *hodginsii* is influenced by climate warming and drought.^[^ ^3^ ^]^ As a long‐lived conifer, *F*. *hodginsii* holds economic value due to its fast‐growing. Especially, *F*. *hodginsii* possesses high medicinally valuable attributed to its secondary metabolites, particularly structurally diverse diterpenoids with antimicrobial activities.^[^ ^4^ ^]^ *F*. *hodginsii* exhibits unique characteristics with its twigs and leaves containing structurally diverse diterpenoids,^[^ ^4^ ^]^ and its roots containing essential oil.^[^ ^5^ ^]^ However, the lack of reference sequences has hindered investigations into the function of bioactive compounds at the molecular level. Therefore, the availability of the *F*. *hodginsii* genome can facilitate molecular studies on gymnosperm plants.

At the early stage, short‐read sequencing was utilized for *de novo* assembly of trees genome, including *Phyllostachys edulis*,^[^ ^6^ ^]^ *Populus euphratica*,^[^ ^7^ ^]^ *Gnetum montanum* ^[^ ^8^ ^]^ and *Picea abies*.^[^ ^9^ ^]^ More recently, long‐reads sequencing has provided significant advantage in assembling complex genome.^[^ ^10^ ^]^ With the availability of long reads platform and analytical tools, the scientific community has reported several assemblies of tree genomes such as *Ginkgo biloba*,^[^ ^11^ ^]^ *Pinus tabuliformis*,^[^ ^12^ ^]^ *Cycas panzhihuaensis*,^[^ ^13^ ^]^ and *Larix kaempferi*.^[^ ^14^ ^]^ The overexploitation of *F*. *hodginsii* has threatened the genetic variation and distribution of this endangered relic gymnosperm. However, a chromosome‐level assembly of *F*. *hodginsii* genome is currently unavailable. Here, we successfully achieved *de novo* whole‐genome assemblies of *F*. *hodginsii*, generating a chromosome‐level reference sequence. Moreover, PacBio Iso‐seq and RNA‐seq data from different tissues of *F*. *hodginsii* provided annotation of TE and coding genes including post transcriptional regulation. Especially, constructing stable genetic transformation system and manipulating genes related to secondary growth are two crucial aspects for exploring the growth mechanism of *F*. *hodginsii*. Thus, BS‐seq, quantitative proteomics, and DRSof xylem and callus revealed the potential regulatory role of DNA methylation and RNA full‐length ratio, which was associated PAL and APA. Finally, we generated single nucleotide polymorphism (SNP) for 14 different cultivars based on the chromosome‐level reference sequences. The chromosome‐level *F*. *hodginsii* genome, along with the epigenetics resource for callus and xylem, will enable *F*. *hodginsii* to serve as a valuable model for investigating callus formation and xylem development at molecular level, facilitating its rational utilization.

## 2 Results

### 2.1 Genome Survey Analysis of *F*. *hodginsii* Characters

**Figure 1:**
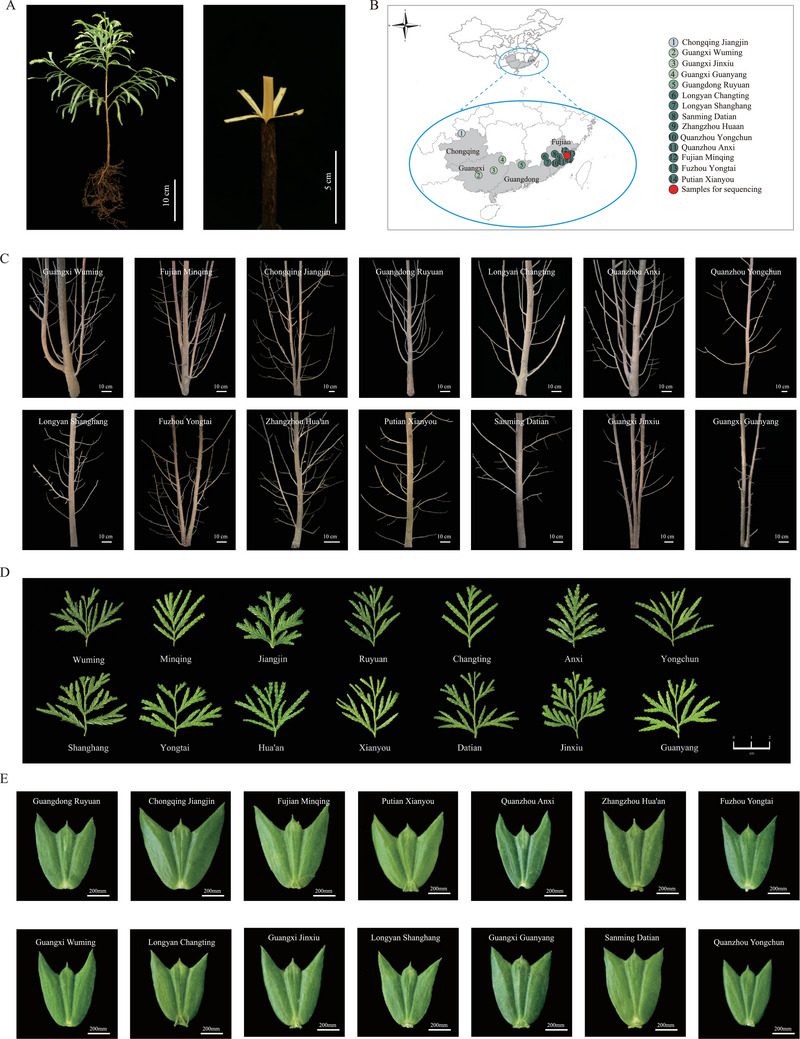
The materials employed for *F. hodginsii* genome sequencing, along with the diverse morphology observed in 14 cultivars. A) xylem samples and genome sequencing utilized material derived from *Fokienia hodginsii*. B) Depicts the general distribution of the 14 different cultivars. The ranges of each circle were approximations aimed at conveying the general distribution of the 14 cultivars rather than detailed distribution. C) Exhibits the varying stem morphology among the 14 cultivars. D) Illustrates the diverse branches morphology found in the 14 cultivars. E) Displays the leaf phenotype of the 14 cultivars.

*F*. *hodginsii*, as a typical gymnosperm species from Cupressaceae, includes xylem that participates in the wood‐forming process (**Figure** **1**A). *F*. *hodginsii* has a widespread distribution in china (Figure 1B). Different cultivars of *F*. *hodginsii* exhibit variations in stem shapes (Figure 1C), branches (Figure 1D), and leaf morphology (Figure 1E). For whole‐genome sequencing, we selected a three‐year‐old *F*. *hodginsii* tree with a height of 3.12 m and a diameter of 2.51 cm at breast height (DBH) from Yongtai County of Fujian province (Figure 1A). Flow cytometry using *Picea abies* as internal reference estimated the genome size of *F*. *hodginsii* to be ≈9.85 G (Figure S1A, Supporting Information). Furthermore, we used a total of 2317255253 paired reads with 150 bp in length from the Illumina platform to further estimate the genome size of *F. hodginsii*. Analysis of the 17‐mer frequency distribution showed a peak depth of 62 and 620402150017 k‐mers (Figure S1B, Supporting Information). Thus, the genome size was estimated to be ≈9984.6 Mb (Kmer‐number/depth), which closely matched the estimation from flow cytometry. The heterozygous ratio and GC content of the *F. hodginsii* genome were estimated to be 0.54% and 35.23%, respectively (Figure S1C, Supporting Information).

### 2.2 *De novo* Assembly and Hi‐C Assisted Genome Assembly of *F*. *hodginsii* Genome

To achieve a high‐quality *de novo* assembly of the *F. hodginsii genome*, we utilized PacBio sequencing to overcome the high level of repetitiveness. The total data of 1199.82 Gb represented ≈120‐fold coverage of the *F. hodginsii* genome. We used FALCON assembler^[^ ^15^ ^]^ for large genome assembly, which generated the initial genome assembly based on the 120‐fold coverage of single‐molecule sequencing (Figure S1D, Supporting Information). The initial PacBio genome assembly resulted in a contig N50 of 3690720 base pairs (Table S1, Supporting Information). The final genome size of *F. hodginsii* was 9.9 Gb. The GC content of the *F. hodginsii* genome is 34.60% (Figure S2A, Supporting Information), consistent with the result from the genome survey. After supplementary assembly with 10X Genomics, the scaffold N50 was 5950457 base pairs, with the largest scaffold reaching 58043276 base pairs (Table S1, Supporting Information).

**Figure 2:**
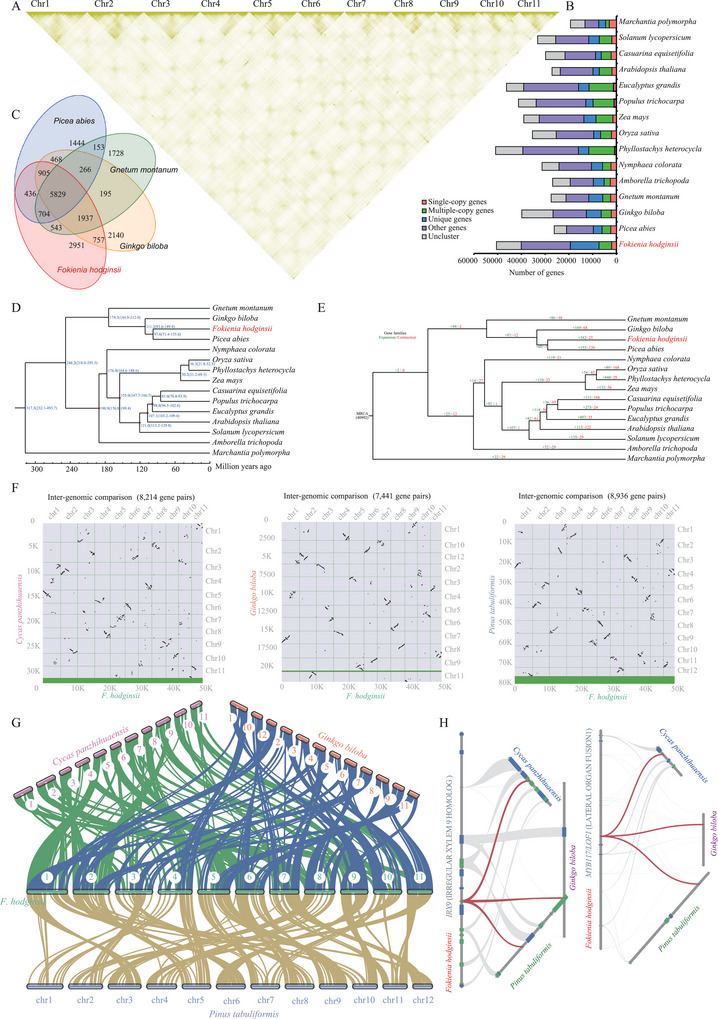
Chromosome‐scale assembly and comparative genomics of *F. hodginsii*. A) The HiC interaction heatmap of 11 *F. hodginsii* chromosomes. B) The distribution of genes in 15 different species. C) Common and unique gene families among 4 different species. D) Estimation of divergence time. E) Expansion and contraction in gene families. The numbers in green and red represent genes family with expansion and contraction, respectively. F) Dot plot illustrating the synteny comparisons between *F. hodginsii* and the three gymnosperms. G) Analysis of macro‐synteny relationships. The green, blue, and yellow lines denote the syntenic relationships of *F. hodginsii* with other gymnosperms. H) Analysis of micro‐synteny. The red lines in the left and right panels indicate homologous genes, respectively. Gray lines denote adjacent micro‐syntenic genes.

To generate a reference‐quality assembly, we utilized Hi‐C data and the ALLHiC algorithm^[^ ^16^ ^]^ to resolve the assembly of the *F. hodginsii* genome. Finally, the Hi‐C incorporated scaffolds into 11 pseudochromosomes (**Figure** **2**A), consistent with published karyotypic studies.^[^ ^17^, ^18^ ^]^ The anchored rate was 99.74%, indicating a high‐quality chromosome‐scale assembly, which generated 9.98G reference‐quantity sequences, with the largest three chromosomes being more than 1G (Figure S2B, Supporting Information). In summary, this study provides a high‐quality reference genome assembly for investigating gymnosperm lineages.

### 2.3 Genome Assembly Evaluation of *F*. *hodginsii*

In accordance with the Earth Biogenome Project (EBP) standards for genome assembly quality control, we performed evaluation on gene completeness assessment, the assembly consensus quality value (QV), and transcript mappability. We performed a BUSCO analysis to assess the completeness of the *F. hodginsii* assembly. The BUSCO (v5.3.2) assessment based on eukaryota_obd10 lineage dataset (Creation date: 2024‐01‐08) revealed that 92.6% and 91.8% of complete BUSCO marker genes in transcriptome and protein model, respectively (Figure S2C, Supporting Information). Additionally, the gene completeness of the *F. hodginsii* genome assembly was evaluated using OMArK,^[^ ^19^ ^]^ which reported a completeness score of 92.88%. OMArK's assessment method is based on the overlap between the query gene set and a conserved ancestral gene set. The identification of 92.88% completeness highlights the high completeness of our assembly and indicates a significant representation of conserved hierarchical orthologous groups.

We further enriched our evaluation about the correctness of the genome assembly using GAEP^[^ ^20^ ^]^ on 10X genomics data. This analysis revealed a robust score of 98.89% for k‐mer completeness. QV represents a log‐scaled probability of error for the consensus base calls, with higher QVs (Q30 corresponds to 99.9% accuracy) indicating more accurate consensus based on Merqury.^[^ ^21^ ^]^ QV scores approaching 40 across all 11 chromosomes indicate high genome assembly quality (Figure S2D, Supporting Information).

The gymnosperm genome is marked by a significant presence of repetitive sequences, with long terminal repeats being the predominant transposable elements.^[^ ^12^ ^]^ Because of the repetitive nature of these elements, assembling them presents a considerable challenge. Our assessment focuses on the continuity of the assembly, utilizing the LTR Assembly Index (LAI) to evaluate assembly quality, divided into three categories: draft (LAI < 10), reference (10‐20), and gold (LAI > 20).^[^ ^22^ ^]^ The LAI values range from 12.66 to 13.93 across each chromosome, indicating a reference assembly quality (Figure S2E, Supporting Information).

We also aligned all RNA‐Seq reads and Nanopore direct RNA sequencing reads to the assembled genome sequence. Over 92% of the RNA‐Seq reads and 96% of the Nanopore long reads could be aligned to the genome, further confirming that the majority of the transcriptome regions were covered in the current genome sequence. Finally, we randomly selected 16 pairs of primers to amplify regions of the *F. hodginsii* genome, followed by conventional Sanger sequencing. Upon comparison with the assembled genome, we found that most regions exhibited over 98% identity (Table S2, Supporting Information). Consistent experimental validation was achieved across all 11 chromosomes, indicating a high degree of reliability for the genome produced in this project.

### 2.4 Annotation of Protein‐Coding Genes and Repetitive Sequences in *F. hodginsii*

In this study, we used transcriptome‐based alignment, *de novo* prediction, and homology‐based searches for the annotation of protein‐coding genes (Figure S3A, Supporting Information). Overall, we obtained 49701, 42093, and 25992 annotated protein‐coding genes from *De novo* prediction, homology search, and transcriptome sequencing, respectively (Figure S3B, Supporting Information). By combining various methods, the EVM annotation pipeline yielded a total of 50521 protein‐coding genes for the *F. hodginsii* genome. The average length of exons and introns was 269 and 7424, respectively. The presence of long introns was similar to that observed in the conifer species *Picea abies*. Furthermore, known protein databases were utilized for gene function annotation through similarity searches of the aforementioned annotated gene structures. Overall, 71.7%, 86.3%, 51.0%, 89.0%, 53.6%, and 67.8% of the genes were assigned putative function through homology searches against Swissprot, Nr, KEGG, InterPro, GO, and Pfam database, respectively (Figure S3C, Supporting Information). In addition to coding genes, we also identified 6897 microRNAs (miRNAs), 3560 transfers RNAs (tRNAs), 1641 ribosomal RNAs (rRNAs), and 1970 small nuclear RNAs (snRNAs) in the *F. hodginsii* genome.

By combining of *de novo* prediction and RepeatMasker annotation, we discovered that 63.11% of the *F. hodginsii* genome was classified as repetitive regions, similar to *Pinus tabuliformis* (69.4%)^[^ ^12^ ^]^ and *Larix kaempferi* (66.8%).^[^ ^14^ ^]^ The percentage of transposable elements (TE) was consistent with that of major gymnosperm lineages. DNA transposons and retrotransposons accounted for 0.41% and 62.29% of the total *F. hodginsii* genome sequence, respectively. Long terminal repeats (LTRs) were most abundant type of TEs, constituting 61.93% of the *F. hodginsii* genome sequences. LTR retrotransposons in *F. hodginsii* exhibited a higher percentage compared to those in *Ginkgo biloba* (52%)^[^ ^11^ ^]^ and *Pinus tabuliformis* (60%).^[^ ^12^ ^]^ We observed two bursts for LTR transposons (Figure S3D, Supporting Information). The initial burst (high k‐values) represented the ancient transposition events, followed by a second burst (middle k‐values) representing the recent transposition events. The burst of DNA transposons displayed low K‐values, representing the most recent copies (Figure S3D, Supporting Information).

### 2.5 Clustering of Ortholog and Evolutionary Analysis

Cluster analysis from 15 sequenced genomes using OthoMCL revealed that these genomes could generate 41001 gene families (Figure 2B). Among these gene families, the 15 sequenced genomes shared a core set of 3465 gene families. When comparing *F. hodginsii* with *Picea abies*, *Gnetum montanum*, and *Ginkgo biloba*, we identified 2951 specific gene families, encompassing a total of 1,4518 genes (Figure 2C). *F. hodginsii* and *Picea abies* were clustered together on a branch (Figure 2D). The divergence time between *F. hodginsii* and *Ginkgo biloba* was estimated to be 111.2 Mya (Figure 2E). Furthermore, we observed that 342 gene family (7405 genes) showed expansion, while 25 gene family (56 genes) exhibited contraction (Figure 2E).

Currently, *G. biloba*,^[^ ^11^ ^]^ *P. tabuliformis*,^[^ ^12^ ^]^ and *C. panzhihuaensis* ^[^ ^13^ ^]^ are three gymnosperms that possess chromosome‐level genomes. In comparative genomic analysis of four representative plant species using OrthoFinder, we identified a total of 185518 genes. Of these, 156414 genes were grouped into orthogroups, constituting 84.3% of the total gene content. Our study delineated 28298 orthogroups, highlighting the extensive conservation and divergence of gene families across these species. Notably, we observed a substantial number of species‐specific orthogroups, totaling 12013, which include 58415 genes, accounting for 31.5% of all analyzed genes. These findings provide crucial insights into the genomic architecture and evolutionary dynamics of these phylogenetically diverse plants, offering a robust framework for further exploration of their unique biological features and evolutionary history.

We conducted a global and local synteny analysis of the aforementioned four species using JCVI.^[^ ^23^ ^]^ The syntenic blocks between *F. hodginsii* and *C. panzhihuaensis*, *G. biloba*, and *P. tabuliformis* revealed 8214, 7441, and 8936 gene pairs, respectively (Figure 2F). We found that several chromosomes of *F. hodginsii* matched to more than one chromosome of other gymnosperms. For example, Chr6 in *F. hodginsii* corresponds to Chr6 and Chr2 in *C. panzhihuaensis*, Chr10 and Chr9 in *G. biloba*, and Chr8 and Chr9 in *P. tabuliformis*. We also observed several similar instances of one‐to‐multiple chromosomes matches *F. hodginsii*, including chr4, chr5, chr7, chr8, chr9, and chr11 (Figure 2G). For wood formation genes, micro‐synteny analysis demonstrated that orthologous pairs involved in wood‐formation gene maintain collinearity gene order between *F. hodginsii* and other gymnosperms. Synteny pattern was also detected for genes associated with transcription factor (Figure 2H).

We have carefully examined the potential whole‐genome duplication (WGD) events in *F. hodginsii*. The peaks for synonymous nucleotide substitutions (Ks) of syntenic paralogous gene pairs in *F. hodginsii* exhibit a weak peak at a median Ks of ≈2.1 (Figure S4A,B, Supporting Information). This suggests a potential ancient whole‐genome duplication (WGD) event, as indicated by the WGDI.^[^ ^24^ ^]^ However, due to the considerable antiquity of this WGD event, the Ks peak signal is attenuated, likely influenced by the age of the event and the subsequent loss of many duplicate genes from the ancient WGD. Additionally, we did not observe any younger Ks peak, suggesting the absence of a recent gymnosperm‐specific WGD in the genome of *F. hodginsii*. MCScanX reported only 0.61% collinear gene (309 genes), further supporting the Ks results and indicating the lack of recent WGD events in the *F. hodginsii* genome.

Furthermore, the median Ks value for orthologous divergence between *F. hodginsii* and both *Cycas panzhihuaensis* and *Ginkgo biloba* is ≈0.8 (Figure S4C,D, Supporting Information). The similarity in Ks values suggests that *F. hodginsii* diverged from these two species at a roughly equivalent time. In contrast, the Ks value between *F. hodginsii* and *P. tabuliformis* is slightly lower, indicating a more recent divergence between *F. hodginsii* and *P. tabuliformis*.

### 2.6 Transcriptome and Post‐Transcriptional Regulation in *F. hodginsii*

Alternative splicing (AS) is a post‐transcriptional event that enhances transcript diversity. To uncover AS events in *F. hodginsii*, we mixed xylem, leaf, branch, and root together and utilized long reads from PacBio Iso‐seq to identify skipped exon (SE), retained intron (RI), alternative 5′ splice site (A5SS), and alternative 3′ splice site (A3SS). RI events were the most prevalent in *F. hodginsii* (Figure S5A, Supporting Information). For example, Alpha galactosidase A exhibited three RI events based on PacBio Iso‐seq long reads (Figure S5B, Supporting Information). These three RIs were located within the translated region, potentially altering the ORF and impacting the function of Alpha galactosidase A. These AS events provide preliminary information for further investigation of post‐transcriptional regulation mechanisms in gymnosperm.

**Figure 3:**
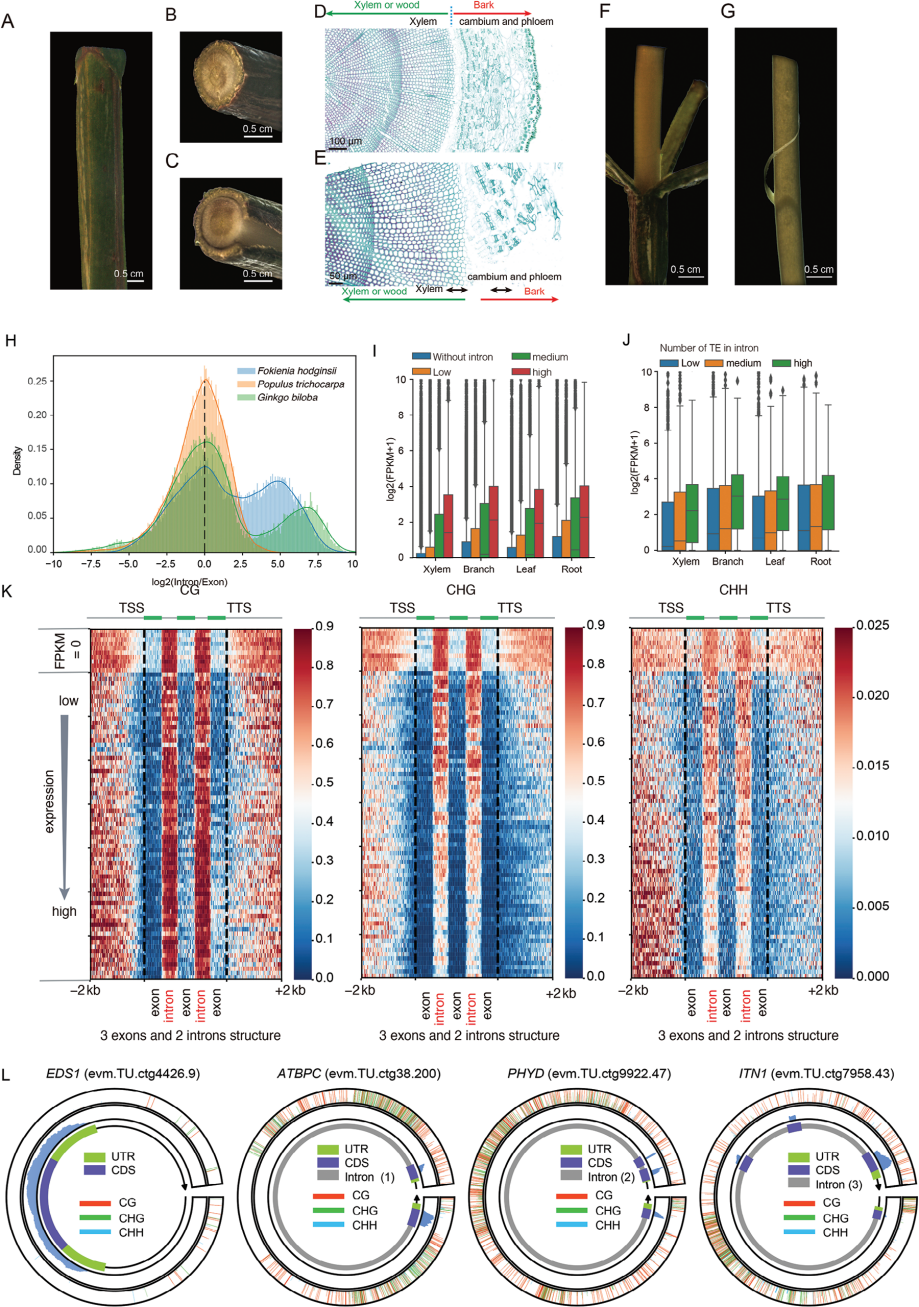
Paraffin‐embedded tissue sections and Transcriptome profile of *F. hodginsii*. A) A segment of the stem from *F. hodginsii* grown in a greenhouse‐grown. B) The cross‐section view of the entire stem. C) The stem showing partial separation of bark from the wood. D) Paraffin‐embedded tissue sections prepared from the stem tissue. E) Stem with bark partially detached from the wood. F) Xylem cells are collected by first removing the bark from the stem. G) The stem surface is scraped with a razor blade to obtain stem strips. H) The distribution of intron/exon length density in *F. hodginsii, Populus trichocarpa*, and *Ginkgo biloba*, respectively. I) Boxplot showing the expression (log2(FPKM + 1)) of genes with different log2(intron/exon) length. The genes were divided into four subsets. One subset consisted of genes lacking introns. The remaining genes retaining introns were classified into three levels (low, medium, and high) based on the log2(intron/exon) value. Each level constituted one‐third of the total number of intron‐retaining genes. J) Boxplot showing the expression (log2(FPKM + 1)) of intron‐including genes with different number of TEs. Genes were grouped into three subsets according to the number of TE, from low to high. K) The distribution of CG, CHG, and CHH methylation levels for genes with three exons. L) From left to right, four cyclic graphs represent genes with one exon, one intron, two introns, and three introns. On the circular diagram, from the innermost to the outermost, they represent gene structure annotation, transcriptome expression signals, and DNA methylation information.

We performed RNA‐Seq analysis for xylem, leaf, branch, and root to calculate gene expression level. We conducted microscopic imaging experiments on paraffin sections. Examination of the sections revealed that the bark contained cambium and phloem tissues, while the xylem was preserved in the wood sections (**Figure** **3**A–E). Therefore, we gently scraped the surface layer of xylem cells from the wood sections to avoid contamination with cambium and phloem tissues (Figure 3F,G). Comparing *F. hodginsii* with the angiosperm (*Populus trichocarpa*), we observed that *F. hodginsii* exhibited longer introns (Figure 3H). The length of introns/exons showed a positive correlation with expression levels (Figure 3I). Long intron has a higher frequency of TE insertion. Thus, we categorized genes with intronic TEs into three categories (low, medium, and high) according to number of TEs. It was evident that genes with TEs in their introns displayed higher expression (Figure 3J). This result suggests that introns including TEs were associated with mRNA expression, which is consistent with a previous study in *Pinus tabuliformis*.^[^ ^12^ ^]^ Whole‐genome Bisulfite sequencing (BS‐Seq) of xylem indeed confirmed high DNA methylation in intron regions (Figure 3K). We did observe a high level of methylation in genes that include introns. Conversely, single‐exon genes displayed low methylation within their gene body regions (Figure 3L).

### 2.7 DNA Methylation Profile of Callus and Xylem in *F. hodginsii*

**Figure 4:**
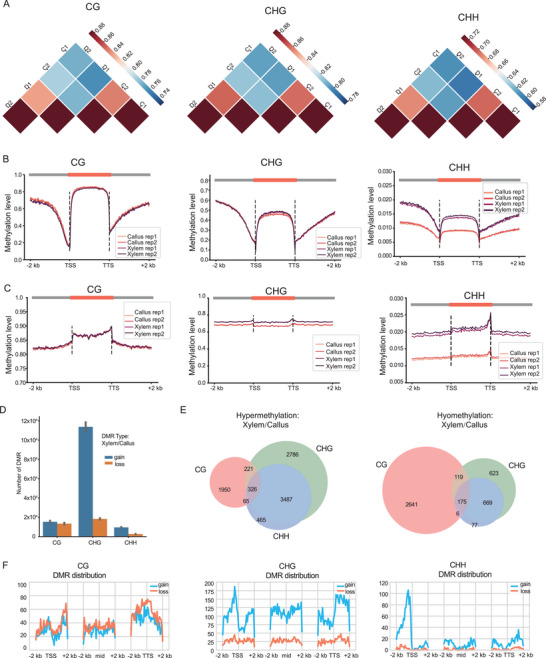
DNA methylation profile of callus and stem‐differentiating xylem from *F. hodginsii*. A) Heatmap presenting Spearman's correlation coefficient among biological repeats and different tissues. B) The profile of DNA methylation in coding genes. C) The profile of DNA methylation in TEs. D) Histogram showing the DMRs between xylem and callus. E) Overlapped genes with DMRs in CG, CHG, and CHH. F) DMR distribution around TSS, TTS, and middle region of transcripts.

It is crucial to investigate gene function in gymnosperm using genetic engineering technology. Callus, as a differentiated tissue type, plays an important role in plant regeneration and Agrobacterium‐mediated stable transformation. We successfully induced callus in *F. hodginsii* and proceeded to investigate DNA methylation in the callus. We used xylem, a differentiated tissue, as a control. The Spearman's correlation coefficient indicated that biological replicates exhibited higher correlation than the two tissue types (callus and xylem) (**Figure** **4**A). The global DNA methylation profile showed that xylem exhibited higher levels of CHG and CHH methylation compared to the callus, both in coding genes (Figure 4B) and TEs (Figure 4C). Furthermore, the analysis of differentially methylated regions (DMRs) also revealed that xylem had a greater number of gains DMRs compared to the callus (Figure 4D). In total, 326 genes with DMRs exhibited overlap among the three types of DMRs (CG, CHG, and CHH). However, most genes only displayed one type of DMRs (Figure 4E). The distribution of DMR indicated their enrichment around both transcription start sites (TSS) and transcription termination sites (TTS) (Figure 4F). The enrichment of DMR in promoters was particularly obvious in CHH type.

### 2.8 Quantitative Proteomics of Callus and Xylem in *F. hodginsii*

**Figure 5:**
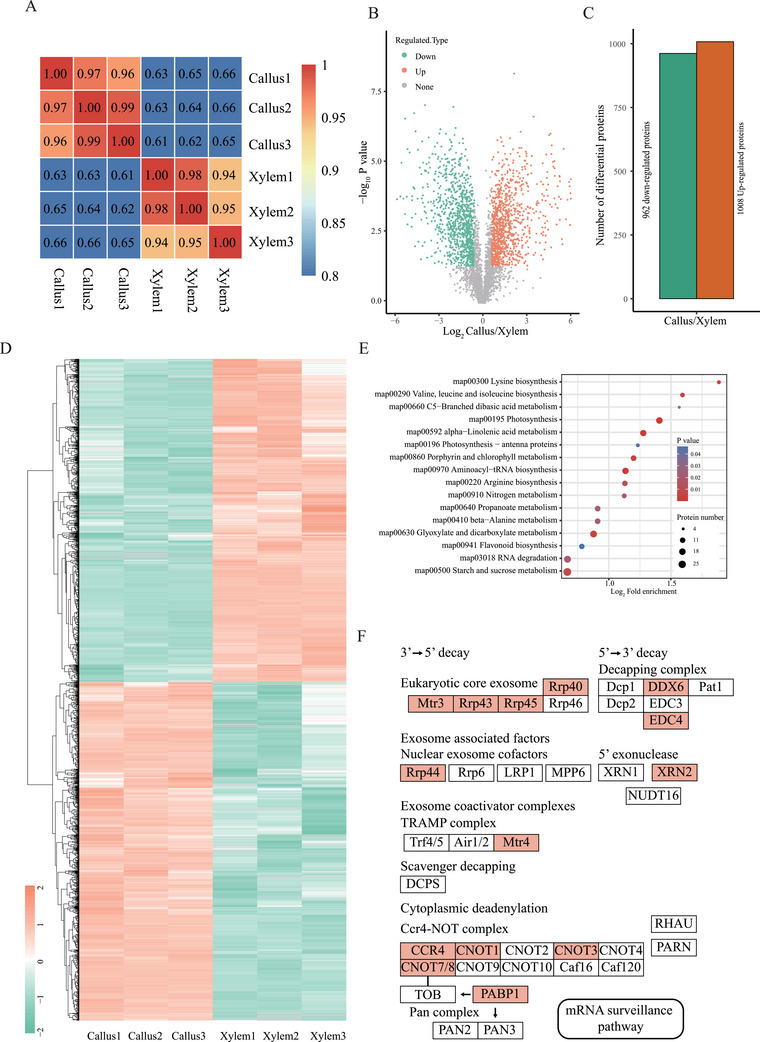
Quantitative proteomics of callus and stem‐differentiating xylem from *F. hodginsii*. A) Heat map presenting Pearson correlation coefficients between all pairwise samples. B) The volcano plot of differential protein expression. C) Histogram showing the number of differentially expressed protein with *P* value < 0.05, FC > 1.5 (Up‐regulation) or FC < 1/1.5 (Down‐regulation). D) Heatmap of 962 down‐regulated proteins and 1008 up‐regulated proteins. E) Bubble chart showing enriched KEGG pathways. F, Schematic diagram showing the differential proteins (red box) in the RNA degradation pathway.

We further investigated the quantitative proteomics of callus and xylem using a 4D Mass Spectrometer. The Pearson correlation coefficients between pairwise samples were consistent with biological and different tissue types (**Figure** **5**A). In this study, we identified differentially expressed proteins based on statistical significance (*P* value < 0.05) versus fold change > 1.5 (green) or < 1/1.5 (red) (Figure 5B). In total, we found 1970 differentially expressed protein (Figure 5C), which exhibited two distinct clusters representing up‐regulation and down‐regulation proteins (Figure 5D). The GO term analysis of the 1008 up‐regulation proteins showed enrichment in negative regulation of photosynthesis, mRNA 3′‐UTR binding, catalytic activity, acting on RNA et al (Figure S6A–C, Supporting Information). The GO term analysis of the 962 down‐regulation proteins exhibited enrichment in plant−type cell wall biogenesis, microtubule binding, and signaling receptor binding et al (Figure S6D–F, Supporting Information). Notably, KEGG pathways analysis revealed the enrichment of RNA degradation (Figure 5E) among the 1008 up‐regulation proteins. This included components of the 5′ → 3′ decay complex (DDX6 and EDC4), 3′ → 5′ decay core exosome (Rrp40/4345 and Mtr3), 5′ exonuclease (XRN2), and CCR4‐NOT complex (CCR4, CNOT1/3/7/8, and PABP1) (Figure 5F).

### 2.9 The Full‐Length Ratio and Poly(A) Length of Callus and Xylem in *F. hodginsii*

**Figure 6:**
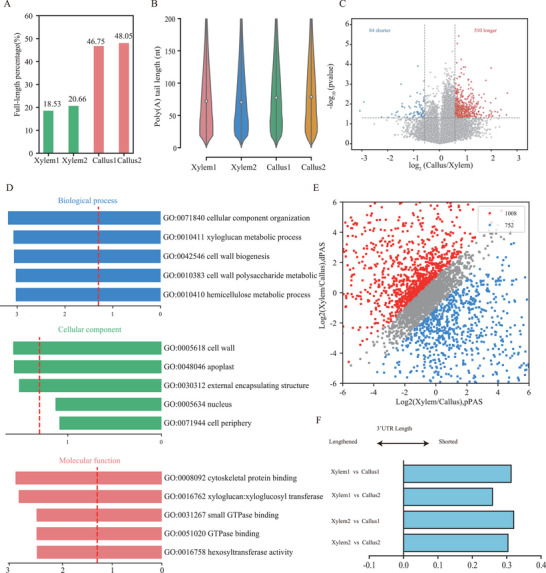
Direct RNA sequencing revealed alterations in the full‐length ratio, PAL, and 3′UTR length in callus and stem‐differentiating xylem from *F. hodginsii*. A) Histogram showing the percentage of the full‐length ratio. B) The violin diagram showing the distribution of PALs. C) The volcano plot of differential PALs. D) Enriched GO terms of genes with longer PALs. E) Scatterplot of differential 3′UTRs. F) The different PALs in callus using the relative expression difference score, which represents the difference in the ratio between dPAS isoform abundance and pPAS isoform abundance between xylem and callus.

Quantitative proteomics revealed differential proteins involved in RNA degradation and mRNA 3′‐UTR binding. Therefore, we performed Nanopore DRS on callus and xylem to reveal the changes in RNA full‐length ratio and APA between the two tissues. DRS revealed that callus had a higher percentage of full‐length reads compared to xylem (**Figure** **6**A). The global distribution of PAL revealed longer PALs in callus than in xylem (Figure 6B), which was consistent with the differential analysis (Figure 6C). Genes with longer PALs in callus exhibited enrichment in cell wall biogenesis, cytoskeletal protein binding, hexosyltransferase activity, et al (Figure 6D). Especially, we observed shorter 3′UTRs in callus due to APA (Figure 6E,F). GO term related with RNA binding was observed in both shorter and longer 3′UTR between callus and xylem (Figure S7, Supporting Information). The changed PALs and 3′UTR length may contribute to the alteration of the full‐length ratio between undifferentiated (callus) and differentiated tissues (xylem). However, further evidence is required to determine whether PALs and 3′UTR lengths are associated with RNA degradation.

### 2.10 Physiological Indicators of Different Cultivars of *F. hodginsii*

**Figure 7:**
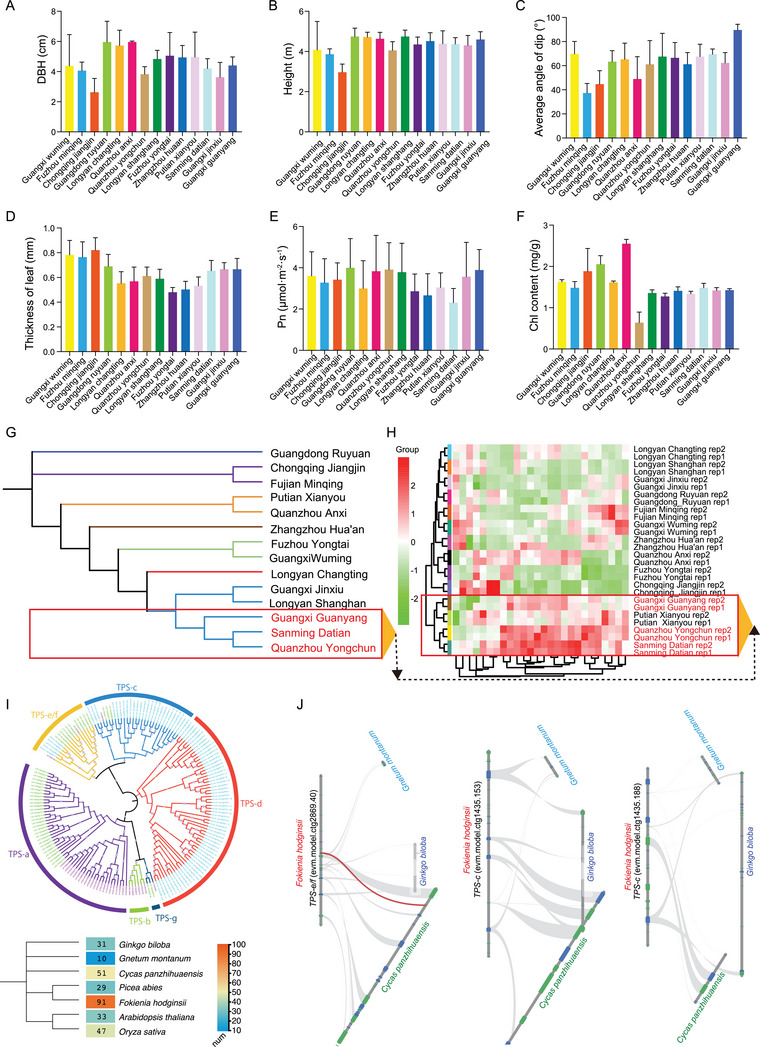
Physiological parameters of *F. hodginsii* in 14 different cultivars. A) Histogram showing the variation in breast height (DBH) among 14 cultivars. B) Histogram showing the variation in stem height among 14 cultivars. C) Histogram showing the variation in the average angle of dip among 14 cultivars. D) Histogram showing the variation in leaf thickness among 14 cultivars. E) Histogram showing the variation in photosynthetic rate among 14 cultivars. F) Histogram showing the variation in chlorophyII content among 14 cultivars. G) Phylogenetic trees of 14 cultivars based on SNP. H) Cluster heatmap is generated using terpene data from a metabonomics analysis. Sample names are listed horizontally, while information about metabolites is presented vertically. Different colors represent values obtained after relative content standardization (red indicates high content, green indicates low content). The clustering lines on the left side of the graph represent sample clustering, and the clustering lines at the top of the graph represent metabolite clustering. I) The upper panel represents the evolutionary analysis of TPS gene in *F. hodginsii*, *Oryza sativa*, and *Arabidopsis thaliana*. Different branch colors represent six different classifications, and gene IDs from different species are differentiated by different colors. The size of the circles in the evolutionary tree represents the bootstrap values. The lower heatmap shows the number of TPS genes in different species. J) Microsynteny analysis between *F. hodginsii* and three gymnosperm species.

The over‐exploitation of *F. hodginsii* has posed a threat to its genetic variation and distribution. In this study, we collected 14 different cultivars of *F. hodginsii* from various regions in China (Figure 1). The leaves and stems (Figure 1) exhibited varying morphologies (Table S3, Supporting Information), which further indicated the genetic diversity and provided preliminary resource for future pan‐genome sequencing of *F. hodginsii*. Physiological measurements were conducted to assess the characteristics of these cultivars (**Figure** **7**). Each cultivar exhibited distinct features. For example, Chongqing Jiangjin had a low DBH, height, and average angle of dip (Figure 7A–C). Fuzhou yongtai had the lowest leaf thickness among the 14 different cultivars (Figure 7D). Sanming datian showed the lowest leaf net photosynthetic rate (Pn) among the 14 cultivars (Figure 7E). Guangzhou yongchun exhibited the lowest chlorophyII (Chl) content among the 14 cultivars (Figure 7F). Furthermore, a phylogenetic analysis based on SNPs of the 14 cultivars revealed seven distinct groups (Figure 7G). We utilized the Genome Analysis Toolkit (GATK) to generate VCF (Variant Call Format) files, which include detailed information about whether each site in the genome is heterozygous or homozygous. We also employed the ASEReadCounter tool to quantify the expression levels of different alleles at specific loci based on RNA‐seq data from the 14 cultivars. This result could provide insights into allele‐specific expression in the different cultivars.

Terpenes are a collective term for polymers and their derivatives of isoprene, with a basic skeleton composed of five carbon units. Terpene compounds are commonly found in *F. hodginsii* organisms and play essential roles. They possess fungicidal properties that enhance a plant's disease resistance, act as allelopathic substances influencing the growth of neighboring plants, serve as effective herbicides, and act as natural insecticides. Furthermore, terpenes exhibit various biological activities such as anti‐inflammatory, anti‐tumor, and antibacterial properties, making them valuable components in pharmaceuticals. Metabolomics analysis of terpenes contributes to the study of their biological functions. The results of phylogenetic evolution showed that the 14 Fujian cypress strains could be classified into distinct groups, such as the Guagnxi Guanyang, Sanming Datian, and Quanzhou Yongchun groups, which also formed a cluster in accordance with the metabonomics results (Figure 7H). Terpene synthases (TPSs) are the key enzymes responsible for the biosynthesis of terpenes. Evolutionary tree analysis reveals that the TPS gene family in *F. hodginsii* can be classified into seven major classes (TPS‐a to TPS‐f), with six of them also present in angiosperms. TPS‐d represents a unique branch exclusive to gymnosperms, with *F. hodginsii* having the highest number of TPS genes among several gymnosperms including *G. biloba*, *G. montanum*, *C. panzhihuaensis*, and *P. abies* (Figure 7I). While we conducted a comparison between *F. hodginsii* and two other gymnosperms, *C. panzhihuaensis* and *G. biloba*, it was very rare to find microsynteny characteristics between the TPS genes of *F. hodginsii* and those of other gymnosperms (Figure 7J). This observation suggests that the physical arrangement of TPS genes within the genomes of gymnosperms appears to lack consistency.

## 3 Discussion

Currently, genome assemblies have been reported for angiosperm species like *P. trichocarpa*,^[^ ^25^ ^]^ *E. grandis*,^[^ ^26^ ^]^ and three representative *Casuarina* species.^[^ ^27^ ^]^ Similarly, several gymnosperm species, such as *G. biloba*,^[^ ^11^ ^]^ *P. tabuliformis*,^[^ ^12^ ^]^ *P. abies*,^[^ ^9^ ^]^ *G. montanu*,^[^ ^8^ ^]^ and *L. kaempferi* ^[^ ^14^ ^]^ have also been extensively studies in the field of forestry. These resources hold significant research value in the field of forestry. However, despite being the largest family within the gymnosperms, Cupressaceae lacks a reported chromosome‐level genome. *F*. *hodginsii*, a member of the Cupressaceae family that survived in southern China and northern Vietnam, holds significant research value due to its diverse diterpenoids with antimicrobial activities.^[^ ^4^ ^]^ However, the genome of *F. hodginsii* had not been reported thus far due to challenges posed by repeat sequences and its large genome size, which have impeded investigations into the growth and development of Cupressaceae. To overcome the disadvantage posed by short reads, we employed PacBio CLR reads to generate chromosome‐level assemblies of the *F. hodginsii* genome, allowing for the annotation of 50521 coding genes. This genome sequencing was conducted before the emergence of PacBio HiFi sequencing technology. Therefore, to assess the quality of genome assembly, we employed several methods for evaluation. Based on the latest lineage dataset, the BUSCO score supports the quality of the genome assembly. This was confirmed by LAI values and mapping transcriptome reads to the *F. hodginsii* genome. This resource of this study greatly facilitates the investigation of gene function in *F. hodginsii*.

Our analysis revealed that a total of 63.11% of the *F. hodginsii* genome sequence consists of DNA transposons and retrotransposons. LTR retroelements accounted for 61.93% of the total *F. hodginsii* genome. We also observed long TE‐including introns in the gene structures of *F. hodginsii*, which were associated with mRNA expression (Figure 3C). Plant genomes generally tolerate TEs within introns, as the TE insertions do not compromise gene function. However, TE insertion can influence the regulation of gene expression through various mechanisms.^[^ ^28^ ^]^ However, there is a significant lack of research on the regulation mechanisms of TEs on gene expression in Cupressaceae. The presence of TEs within introns is widespread in gymnosperms. Therefore, investigating the potential mechanisms by which introns regulate expression through interplay with TE and DNA methylation would be intriguing.

We utilized the Centromics^[^ ^29^ ^]^ to identify centromeres on *F. hodginsii* chromosomes. Additionally, we plotted gene density and transposon densities (including DNA, LINE, and LTR) across the chromosomes to further assist in identifying centromeres regions (Figure S8, Supporting Information). However, for more precise identification of centromeres regions in future studies, the inclusion of additional ChIP‐seq data for CENH3 could be advantageous.

Investigating gene function in gymnosperms using genetic engineering technology poses challenges. In the future, we plan to develop Agrobacterium‐mediated transformation in *F. hodginsii*, in conjunction with epigenetic and genomic data in this study. Currently, we have successfully induced calluses in *F. hodginsii* by utilizing young shoots as explants. Therefore, we used callus and xylem as materials to explore DNA methylation regulation and post‐transcriptional regulation, including RNA degradation, PALs, and APA. Quantitative proteomics revealed the up‐regulation of RNA degradation component in callus, such as the 5′ → 3′ and 3′ → 5′ decay complex, 5′ exonuclease, and CCR4‐NOT complex (Figure 5F). Based on the proteomics result, we expected higher RNA degradation in callus. However, to our surprise, we found that callus presented a higher full‐length ratio compared to xylem. Additionally, we observed higher PALs in callus, which might partially counteract RNA degradation. Moreover, we also observed shorter 3′UTR in callus caused by APA. Isoforms with shorter 3′UTR length in callus might also display different degradation ratios due to shorted isoforms excluding regulated motif in the 3′UTR. However, further evidence is required to investigate the association between RNA degradation and PAL/3′UTR length.

Altogether, we successfully generated a high‐quality reference genome sequence for *F. hodginsii*, providing preliminary insights into this Cupressaceae species. This resource will be essential for subsequent investigations into the genes and their functional significance. Furthermore, transcriptome analysis and epigenetic profiling were conducted for xylem and callus to explore potential association between gene expression and epigenetic regulation. Finally, we examined different cultivars from various geographic location in China. All results in this study offer valuable resources for investigating biological functions at the molecular level within the Cupressaceae family.

## 4 Experimental Section

### Sample Collection and DNA Extraction

In this study, three‐year‐old *F. hodginsii* (3.12 m in height, 2.51 cm in DBH) was collected at Yongtai County, Fujian province (E119°140; N26°050), for genome sequencing. The collected material was immediately frozen in liquid nitrogen, and genomic DNA extraction was performed using the plant mini kit (Qiagen, Hilden, Germany). Qubit fluorometer (Thermo Fisher Scientific) and Agarose gel electrophoresis were used to quantify DNA concentration and confirm high integrity.

For RNA sequencing, five different tissues were collected, including xylem, leaf, branch, root, and callus. The callus of *F. hodginsii* was induced from young shoot using MS medium supplemented with 6‐BA at 2.0 mg⋅L^−1^, 2,4‐D 1.0 mg⋅L^−1^, and KT 0.5 mg⋅L^−1^. For Sanger sequencing validation, 2 µL of the PCR products were subjected to agarose gel electrophoresis to verify the size of the amplified fragments against the expected target. The confirmed PCR products were then sequenced using the Sanger method on an ABI 3730xl Genetic Analyzer (Applied Biosystems) to ensure high fidelity and accuracy of the assembled genome.

### Paraffin‐Embedded Tissue

The tissue was ideally thinned to 2–3 mm and placed in a dehydration chamber. The chamber was then submerged in a plant softening solution, sealed, and incubated in a 55 °C oven for softening. The softening solution should be changed every 7 days, and the softening progress should be assessed weekly. Softened plants were rinsed under running water for 30 min and subsequently soaked in 15% ethanol for 2 h. The tissue was then dehydrated in a vacuum tissue dehydrator through a series of ethanol concentrations: 30%, 50%, 75%, 85%, 90%, 95%, and 100%, each for specified durations, followed by xylene treatment and embedding in paraffin. Melted wax was poured into embedding frames, and the tissue was positioned before the wax solidifies. Labels were affixed as necessary. The wax blocks were cooled at −20 °C, then sectioned into 5 µm slices using a paraffin microtome. Sections are floated on warm (40 °C) water, transferred onto slides, and baked at 60 °C. After baking, the slides were stored at room temperature.

Slides were dewaxed using two changes of an environmentally friendly dewaxing solution, each for 20 min, followed by two changes of pure ethanol for 5 min each, and a 5‐min rinse in 75% ethanol. The slides were then rinsed in tap water. Sections are stained in Safranin‐O solution for 2 h and rinsed with tap water. They were then briefly treated with 50%, 70%, and 80% ethanol. Staining is carried out with plant solid green solution for 6–20 s, followed by dehydration in anhydrous ethanol. Sections underwent three changes of xylene for 5 min each and were then mounted with neutral gum. Finally, sections were examined under a microscope, images were captured, and analysis was conducted.

### Genome Survey Based on Illumina DNA Sequencing

Illumina TruSeq Nano DNA libraries with 300 bp insert sizes were constructed and sequenced using Illumina HiSeq X Ten sequencers. The surveyed genome was generated using SOAPdenovo^[^ ^30^ ^]^ with a kmer of 41 for assembly. The genome size of *F. hodginsii* was estimated based on 17‐kmer depth analysis using Jellyfish.^[^ ^31^ ^]^

### PacBio DNA Sequencing and *De novo* Assembly

PacBio sequencing libraries with an insert size of 20 kb were constructed using SMRTbell template Prep Kits. The *F. hodginsii* genomic DNA was sheared into ≈20 kb fragments. After a series of process, including damage repair, end repair, blunt‐end adaptor ligation, and size selection, the PacBio DNA libraries were sequenced on the PacBio Sequel instrument with P6‐C4 sequencing reagent using CLR sequencing mode. For genome assembly, the FALCON assembler^[^ ^15^ ^]^ and performed error correction with Quiver were utilized,^[^ ^32^ ^]^ followed by genome assembly improvement with Pilon.^[^ ^33^ ^]^ Finally, purge_haplotigs^[^ ^34^ ^]^ was used to polish the genome assembly and reduce duplication.

### Construction of Hi‐C Libraries and Bioinformatic Analysis of Hi‐C

Previous method^[^ ^35^ ^]^ was followed to construct Hi‐C libraries for *F. hodginsii*. Briefly, the leaves of *F. hodginsii* were fixed with a 1% formaldehyde solution. After fixation, the leaves were incubated in MC buffer. The fixed tissue was then resuspended in a nuclei isolation buffer after homogenizing with liquid nitrogen. Chromatin was digested with HindIII restriction enzyme (NEB) at 37 °C for 16 h. After DNA end labeling with biotin and DNA ligation with T4 DNA ligase (NEB), proteinase K was added to reverse cross‐linking before purifying DNA fragments, which was further fragmented to a size of 300–500 bp size for Hi‐C library construction. Finally, paired‐end Hi‐C reads were obtained using the Illumina Hiseq X Ten sequencer. The Hi‐C clean reads were aligned to the *F. hodginsii* assembly using HiCUP.^[^ ^36^ ^]^ The filter data from HiCUP output were used as input data for ALLHiC^[^ ^16^ ^]^ to reconstruct each chromosome with default options.

### Structure Annotation and Repeat Annotation

Three methods were employed for structural annotation: transcriptome‐assisted prediction, *de novo* prediction, and homology prediction. For transcriptome‐based prediction, the RNA‐Seq reads were aligned to the *F. hodginsii* genome using HISAT^[^ ^37^ ^]^ with default option. The aligned reads were then used as input for Stringtie^[^ ^38^ ^]^ to generate reference‐based transcript assemblies, utilizing default parameters. Additionally, five tools were adopted for *de novo* predictions, including Augustus,^[^ ^39^ ^]^ Geneid,^[^ ^40^ ^]^ Genescan, GlimmerHMM, and SNAP, all with default option. For homolog prediction, TBLASTN (v2.2.26; E‐value ≤ 1e−5) was employed to identify homologous proteins from the Ensembl database, and gene structures were generate using GeneWise (v2.4.1) software.^[^ ^41^ ^]^ Finally, PASA^[^ ^42^ ^]^ was adopted to generate non‐redundant gene annotation by merging genes evidence from above three distinct methods. Gene function was determined by identifying the best match with BLASTP with an E‐value threshold of ≤ 1e−5) against Swiss‐Prot^[^ ^43^ ^]^ and DIAMOND.^[^ ^44^ ^]^ Protein domains were identified by InterProScan.^[^ ^45^ ^]^ Gene Ontology (GO) IDs were assigned for each annotated gene based on the corresponding InterPro entry.

Both *de novo* search and homology alignment methods were utilized for repetitive sequence annotation. For *de novo* prediction of repetitive elements, LTR_FINDER,^[^ ^46^ ^]^ RepeatScout,^[^ ^47^ ^]^ and RepeatModeler^[^ ^48^ ^]^ were employed with their default parameters. Subsequently, RepeatMasker software^[^ ^49^ ^]^ was utilized to predict homologous TE using Repbase database^[^ ^50^ ^]^ with default parameters. Additionally, Tandem Repeats were annotated using Tandem Repeats Finder program.^[^ ^51^ ^]^ To evaluate the integrity of the genome assembly with respect to repetitive sequences, the continuity of assembly was gauged using the LTR Assembly Index.^[^ ^22^ ^]^

### Evolution and Synteny Analysis

MUSCLE^[^ ^52^ ^]^ was used to cluster gene families and construct a phylogenetic tree using RAxML^[^ ^53^ ^]^ with the maximum likelihood method. The divergence time was estimated using MCMCTREE (v4.4) from the PAML package.^[^ ^54^ ^]^ Finally, synteny inference was performed using JCVI utility libraries.^[^ ^55^ ^]^

### RNA‐Seq Libraries Construction and Bioinformatics Analysis

Various tissues were collected, including xylem, callus, leaf, branch, and root, for dUTP strand‐specific libraries. Total RNAs were isolated using the RNAprep Pure Plant Kit. Libraries were construction using the dUTP method, and samples with RIN values greater than eight were used. The libraries were sequenced on the Illumina NovaSeq platform, generating 150‐nt paired‐end reads. The resulting FASTQ files were aligned to the assembled *F. hodginsii* genome using HISAT2 (Version:2.1.0)^[^ ^37^ ^]^ with the default options. The alignment files were filtered, and only uniquely mapped reads were used for featureCounts^[^ ^56^ ^]^ to count pairs of reads that mapped to annotated gene regions. DEseq2^[^ ^57^ ^]^ was used to analyze differentially expressed genes, with a cutoff of FDR < 0.05 and fold change > 2. For GO enrichment analysis, clusterProfiler package was utilized.^[^ ^58^ ^]^

### PacBio Isoform Sequencing and Bioinformatics Analysis

All the samples were pooled into a single library for PacBio full‐length isoform sequencing and constructed Iso‐seq libraries using the method described previously.^[^ ^35^ ^]^ Briefly, magnetic beads with Oligo (dT) were used to enrich poly(A) RNAs, which were then transferred into cDNA. Following the completion of PacBio Iso‐seq library construction, sequencing was performed using the PacBio Sequel sequencing system. For Iso‐seq bioinformatics analysis, SMRT Link, IsoSeq v3, and BAM2fastx tools were utilized to generate highly accurate consensus sequences, remove poly(A) tail, detect concatemers, and convert BAM files into FASTA format. To correct the Iso‐seq long reads, RNA‐Seq reads with LoRDEC were used.^[^ ^59^ ^]^ The corrected long‐reads were aligned to the *F. hodginsii* genome using minimap2.^[^ ^60^ ^]^ Finally, S events were identified based on the aligned long reads using PRAPI^[^ ^61^ ^]^ with the default options.

### Whole‐Genome Bisulfite Sequencing Data Processing and Analysis

The BS‐seq libraries and analysis were constructed based on the method described previously.^[^ ^62^ ^]^ Briefly, bisulfite conversion was performed on genomics DNA from xylem and callus using the EZ DNA Methylation‐Gold kit, following the manufacturer's protocol. Subsequently, paired‐end reads were generated with two biological repeats using Illumina Novaseq 6000 platforms. For the bioinformatics analysis, the *F. hodginsii* genome was indexed using the bismark_genome_preparation from the bismark software (Krueger and Andrews, 2011). Additionally, the bismark_methylation_extractor tool was utilized to obtain DNA methylation levels based on CX report file.

### 4D Mass Spectrometer of Xylem and Callus

Callus and xylem samples from *F. hodginsii*, including three biological repeats, were ground into powders with liquid nitrogen. The powders were then sonicated three times in lysis buffer (10 mm Tris‐HCl (pH 8.0), 5 mm EDTA, 1% (w/v), SDS, 8 m urea, and 20 mm dithiothreitol) using high intensity ultrasonic processor. After adding an equal volume of Tris‐saturated phenol, the mixture was vortexed and centrifuged for 10 min at 4 °C and 5 000 g. Next, ammonium sulfate‐saturated methanol was added to precipitate the upper phenol phase and incubated it at −20 °C for 6 h. The supernatant was discarded after centrifugation at 4 °C for 10 min to obtain the remaining precipitate, which was further washed with pre‐cooled methanol and acetone. The concentration of purified proteins was measured using the BCA protein assay. The 4D quantitative proteomics analysis was performed using a timsTOF Pro mass spectrometer. The mass spectral data was processed using MaxQuant,^[^ ^63^ ^]^ specifying Trypsin/P as the cleavage enzyme and allowing for up to 2 missing cleavages. The false discovery rate (FDR)‐adjusted value was required to be < 1%.

### Nanopore Direct RNA Sequencing Data Processing and Analysis

RNAs from xylem and callus, with two biological replicates were directly sequenced using PromethION platforms (R9.4.1 flow cell), which recorded the current signal generated by RNA molecules passing through the nanopore. The raw electrical signal from xylem and callus was converted into FASTQ sequence using Guppy v3.6.1 (https://github.com/nanoporetech/rerio). The FASTQ files were then converted into FASTA format and corrected using LoRDEC^[^ ^59^ ^]^ based on RNA‐Seq reads obtained from Illumina sequencing. The corrected long reads were aligned to the *F. hodginsii* genome using minimap2.^[^ ^60^ ^]^ Full‐length reads were defined as those exhibiting completeness based on annotated translation start sites, following a previous method.^[^ ^64^ ^]^ The remaining reads that did not meet the full‐length criteria were classified as non‐full‐length reads.

### Estimation of Poly(A) Tail Length and Polyadenylation Sites

PAL was determined based on the raw current signal of each read using nanopolish (https://github.com/jts/nanopolish). Only reads with a QC tag of “PASS” in the nanopolish report were included in the downstream analysis. To assign each read to an annotated gene, the long‐read mode was employed from featureCounts.^[^ ^56^ ^]^ Only reads labeled as “Assigned” and a QC tag of “PASS” were used for further analysis. The PAL for each gene was defined as the median length of all reads associated with that gene. Genes showing a p‐value < 0.05 and a fold change > 1.5 between xylem and callus were considered as differential PAL genes.

The poly(A) site of each read (PAS) was estimated based on its coordinate position, which was aligned to *F. hodginsii* genome. PASs located within 24 nt of each other were grouped into the same cluster. From each cluster, the PAS with the highest abundance and support from at least three DRS reads was selected as the reporting site to ensure high reliability. The number of PAS reads was normalized using RPM based on the total number of raw reads assigned to a gene. For APA analysis in 3′ UTR, the two most abundant PAS clusters were considered on the most 3′ exon. Genes exhibiting a relative abundance (|log2(dPAS1/dPAS2) – log2(pPAS1/pPAS2)|) change >30% were considered to have significant alterations in their 3' UTR length.

### Investigation of Growth Difference and Genetic Diversity of *F. hodginsii*

In this study, 14 different cultivars were collected to investigate their growth and photosynthetic characteristics. These characteristics included DBH, height, average angle of dip, thickness of leaf, Pn, and Chl content. Additionally, RNA‐Seq analysis was performed on the 14 different cultivars, with two biological replicates, to identify SNP. The SNP information for the 14 different cultivars was extracted from Variant Call Format (VCF) files.

### Metabolome for 14 Different Cultivars

In the preparation of biological samples of 14 different cultivars with two biological repeats, vacuum freeze‐dryer was employed to freeze‐dry the samples. Subsequently, the freeze‐dried samples were processed using mixer mill for a duration of 1.5 min at a frequency of 30 Hz. To initiate the extraction process, 100 mg of the lyophilized powder was dissolved in 1.2 mL of a 70% methanol solution. The mixture was vortexed for 30 s at 30‐min intervals, totaling six cycles. Afterward, the sample was placed in a refrigerator at 4 °C overnight. Following this, the extracts underwent centrifugation at 12 000 rpm for 10 min, and the resulting extracts were filtered through a 0.22 µm pore size SCAA‐104 filter before being subjected to UPLC‐MS/MS analysis.

Sample extracts were analyzed using a UPLC‐ESI‐MS/MS system, comprising a UPLC (SHIMADZU Nexera X2) and an MS (Applied Biosystems 4500 Q TRAP). The mobile phase consisted of solvent A (pure water with 0.1% formic acid) and solvent B (acetonitrile with 0.1% formic acid). The analysis employed a gradient program, starting with 95% A and 5% B. Over a 9‐min period, a linear gradient to 5% A and 95% B was applied, and this composition was maintained for 1 min. Subsequently, a 95% A and 5% B composition was achieved within 1.10 min and held for 2.9 min. The flow rate was set at 0.35 mL per minute, and the column oven was maintained at 40 °C. Each injection consisted of 4 µL of the sample. The effluent was directed to an ESI‐triple quadrupole‐linear ion trap (QTRAP)‐MS. LIT and triple quadrupole (QQQ) scans were conducted on an AB4500 Q TRAP UPLC/MS/MS System equipped with an ESI Turbo Ion‐Spray interface, operating in both positive and negative ion modes.

### Data Access

Raw data in this study has been deposited in NCBI Sequence Read Archive (SRA) under BioProject PRJNA914999. DNA methylation data (BS‐seq) and RNA sequencing data (dUTP‐RNA‐seq and Nanopore RNA sequencing) were deposited in the National Genomics Data Center (NGDC) under accession number PRJCA025353. Furthermore, the genome assembly and annotation were uploaded to figshare (https://doi.org/10.6084/m9.figshare.26064412.v1).

### Compliance with Ethics Requirements

There was no use of any animals or human patients in this study.

## Conflict of Interest

The authors declare no conflict of interest.

## Author Contributions

J.R. and Y.Z. contributed equally to this work. Y.Z. and L.G. are co‐corresponding authors. Y.Z. and L.G. conceived and designed the research and coordinated the project. J.R., Y.G., L.F., Z.D., Y.P., B.L., L.C., T.H., L.C., and J.Y., managed the field work and performed the experiments. Z.Z., J.Z., T.H., and M.Z. performed the bioinformatics work. J. R., Y.G., H.Z., and L.G. analyzed the data and prepared the figures. Y.Z., J.Y., and L.G. wrote the manuscript. All authors read and approved the manuscript.

## Supporting information

Supporting Information

## Data Availability

The data that support the findings of this study are available from the corresponding author upon reasonable request.
