# Supplementary material for: De novo Whole‐Genome Assembly of the 10‐Gigabase Fokienia Hodginsii Genome to Reveal Differential Epigenetic Events Between Callus and Xylem
Source: Adv Sci (Weinh). 2024 Sep 4;11(40):2402644. doi: 10.1002/advs.202402644 (PMC11516051; doi:10.1002/advs.202402644)
Supplement: Supplementary file 1 — Supporting Information [file ADVS-11-2402644-s001.docx]

Table S1. N50 Values of Contig/Scaffold Based on PacBio, 10X, and Hi-C Assisted Genome Assemblies

|  | PacBio-based | 10X assisted Hi-C assisted | | | |
| --- | --- | --- | --- | --- | --- |
|  |  | Contig | Scaffold | Contig | Scaffold |
| Total | 9,994,628,482 | 9,994,628,482 | 10,007,846,207 | 9,994,628,482 | 10,008,735,707 |
| Max | 38,592,460 | 38,592,460 | 58,043,276 | 38,541,900 | 1,151,609,127 |
| N50 | 3,690,720 | 3,690,720 | 5,950,457 | 3,446,244 | 901,083,726 |
| N60 | 2,958,085 | 2,958,085 | 4,632,157 | 2,690,687 | 866,474,680 |
| N70 | 2,229,369 | 2,229,369 | 3,459,608 | 1,989,324 | 847,101,581 |
| N80 | 1,540,556 | 1,540,556 | 2,400,244 | 1,320,196 | 784,860,141 |
| N90 | 811,047 | 811,047 | 1,340,949 | 625,914 | 772,718,104 |

Table S2. Primer Design, Sanger Sequencing, and Comparative Analysis with Genomic Sequences

|  | Validation by Sanger Sequencing | | |
| --- | --- | --- | --- |
| Chromosome | Left primer | Right primer | % Identity |
| Hic_asm_0 | GGGAGGACTGTGGACTTCAA | CCATGGCTGTTGGTGATTCC | 99.223 |
| Hic_asm_0 | ACCATGCCCCTTTCGTATCT | AGAGCCACAATTTGCAAGGG | 99.566 |
| Hic_asm_1 | ATCTTGCTGGTGACCCTTGA | CTGGCGGCTTCTACTCTACA | 99.434 |
| Hic_asm_1 | GCAGTGATGATGGTGTGGAC | TCCATCCCCATTCACTTGCT | 99.384 |
| Hic_asm_2 | TTGGAGGCGGGAACAATTTC | TGGCACTCTCTTGGAACCAT | 99.563 |
| Hic_asm_2 | GAGGGCAGAGCAGACCATTA | TGAACTCCCCTCGAAAGCAT | 98.004 |
| Hic_asm_3 | CAAGGGCGTTCTCACAACAA | ACAGATCTACGGGCCTCCTA | 99.618 |
| Hic_asm_4 | GAGCAGCAGTGTCGGTAATG | TCGGCGGAATCTCATTAGCT | 96.980 |
| Hic_asm_5 | TACTACCCATCCAGACCCGA | TCACATCGGCATTGCGAATT | 99.634 |
| Hic_asm_6 | TGCTTTACCTGTTCCCACCA | GAGGCCGGATACATGATTGC | 98.848 |
| Hic_asm_7 | TTCTCAGGCATCCTCACGAA | TCTTCTTGGAGCCCTTTGGA | 99.252 |
| Hic_asm_8 | AAATGGCTCTGTTTCTCCGC | GGTTCCAATGATGTAGCGCA | 99.433 |
| Hic_asm_8 | CAGAAGCTGTGCCATGTTGA | CCCAGGAGCAGAACAAACAC | 98.855 |
| Hic_asm_9 | TGGCACTGGACAGGATCAAT | GGCCATACCCAGACTGAGTT | 99.287 |
| Hic_asm_9 | GCCATGGACTTGCTAAGCTC | AAACCCCTCACCTCATCCAG | 99.219 |
| Hic_asm_10 | GCCTGGAGTAGAGGGATCAC | TTGCCACAGATCTCAGGGAG | 99.807 |

Table S3. Physiological parameters of 14 cultivars of *F. hodginsii*

| Source  ID | Origin | Physiological Parameters | | | |  |  |  |
| --- | --- | --- | --- | --- | --- | --- | --- | --- |
|  |  | Specific Leaf Weight (SLW) | Specific Leaf Area (SLA) | Leaf Thickness | Diameter at Breast Height (DBH) | Height (m) | Average Crown | Average Branch Angle |
| L1 | Guangxi_Wuming | 0.0031 | 318.3428 | 0.7822 | 4.38 | 4.08 | 1.015 | 69.7933 |
| L4 | Fujian_Minqing | 0.0029 | 351.3161 | 0.7644 | 4.06 | 3.86 | 1.135 | 37.302 |
| L5 | Chongqing_Jiangjin | 0.0032 | 312.1521 | 0.8211 | 2.6333 | 2.9667 | 0.8333 | 44.7411 |
| L7 | Guangdong_Ruyuan | 0.0031 | 325.034 | 0.6894 | 5.96 | 4.74 | 1.28 | 63.3987 |
| L8 | Longyan_Changting | 0.0029 | 349.304 | 0.553 | 5.72 | 4.72 | 1.165 | 65.2733 |
| L9 | Quanzhou_Anxi | 0.0031 | 319.9205 | 0.5689 | 5.9667 | 4.6333 | 1.225 | 48.9278 |
| L11 | Quanzhou_Yongchun | 0.0032 | 308.976 | 0.6111 | 3.825 | 4.05 | 1.1375 | 61.1225 |
| L13 | Longyan_Shanghang | 0.0031 | 319.0704 | 0.5894 | 4.84 | 4.74 | 1.18 | 67.5467 |
| L14 | Fuzhou_Yongtai | 0.0029 | 351.4977 | 0.48 | 5.05 | 4.35 | 1 | 66.5292 |
| L15 | Zhangzhou_Hua'an | 0.0026 | 385.0787 | 0.5028 | 4.94 | 4.52 | 1.205 | 61.18 |
| L18 | Putian_Xianyou | 0.0032 | 313.8245 | 0.5322 | 4.96 | 4.38 | 1.17 | 67.52 |
| L19 | Sanming_Datian | 0.0038 | 269.0995 | 0.655 | 4.2 | 4.3667 | 1.275 | 69.4667 |
| L22 | Guangxi_Jinxiu | 0.0077 | 260.0208 | 0.6667 | 3.625 | 4.3 | 1.1125 | 62.2583 |
| L23 | Guangxi_Guanyang | 0.0031 | 325.962 | 0.6661 | 4.4 | 4.6 | 1.195 | 89.6433 |


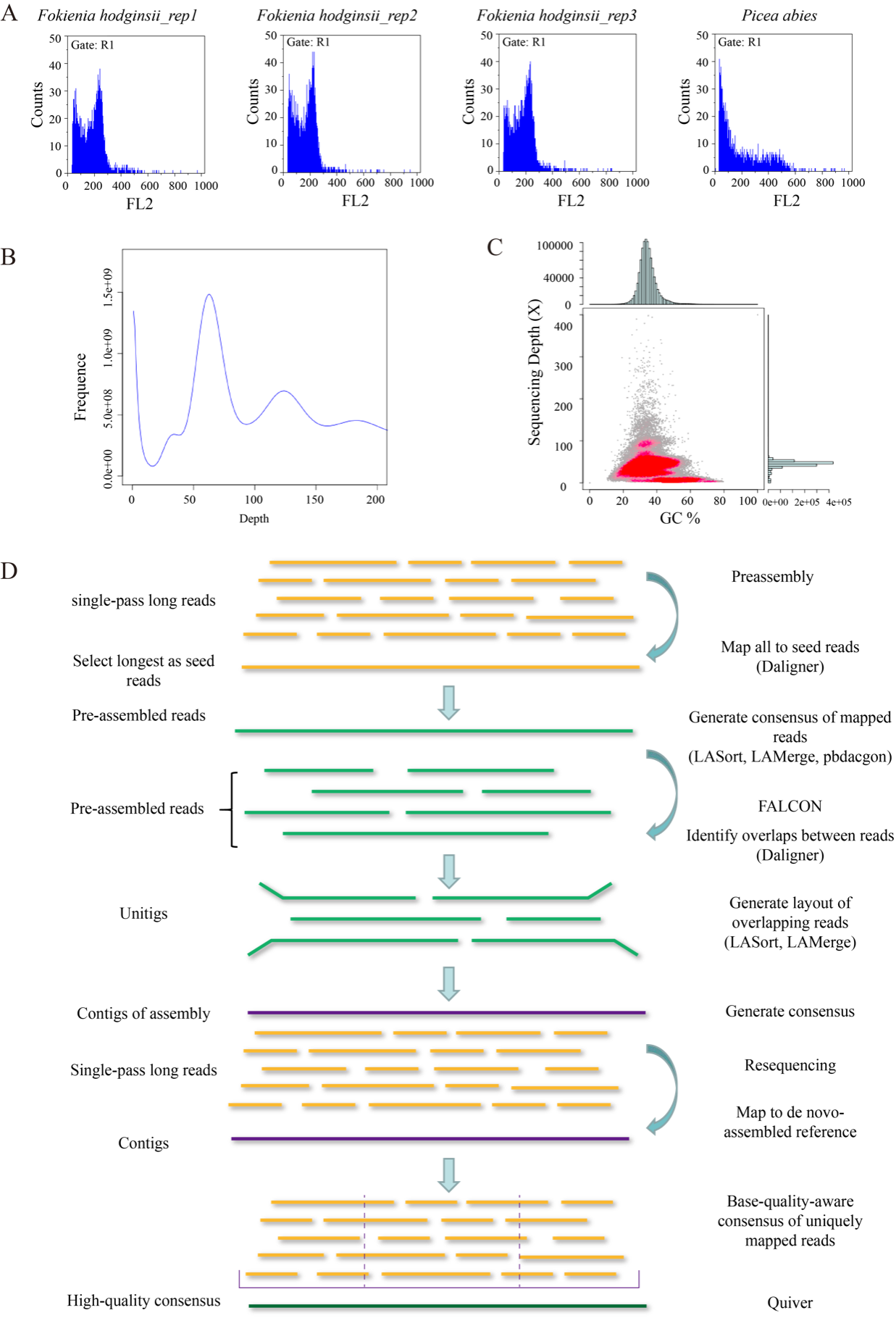


**Supplementary Figure S1**. Flow cytometry and genome survey conducted on the genome of *F. hodginsii* and the process of genome assembly*.* (A) Comparison of flow cytometry analysis between *F. hodginsii* and *Picea abies*. (B) K-mer frequency distribution observed in the genome survey of *F. hodginsii.* (C) The scatter plot presenting the GC content distribution under different sequencing depth based on short reads from Illumina platform. (D) Flowchart illustrating the process of genome assembly for *F. hodginsii*.

*
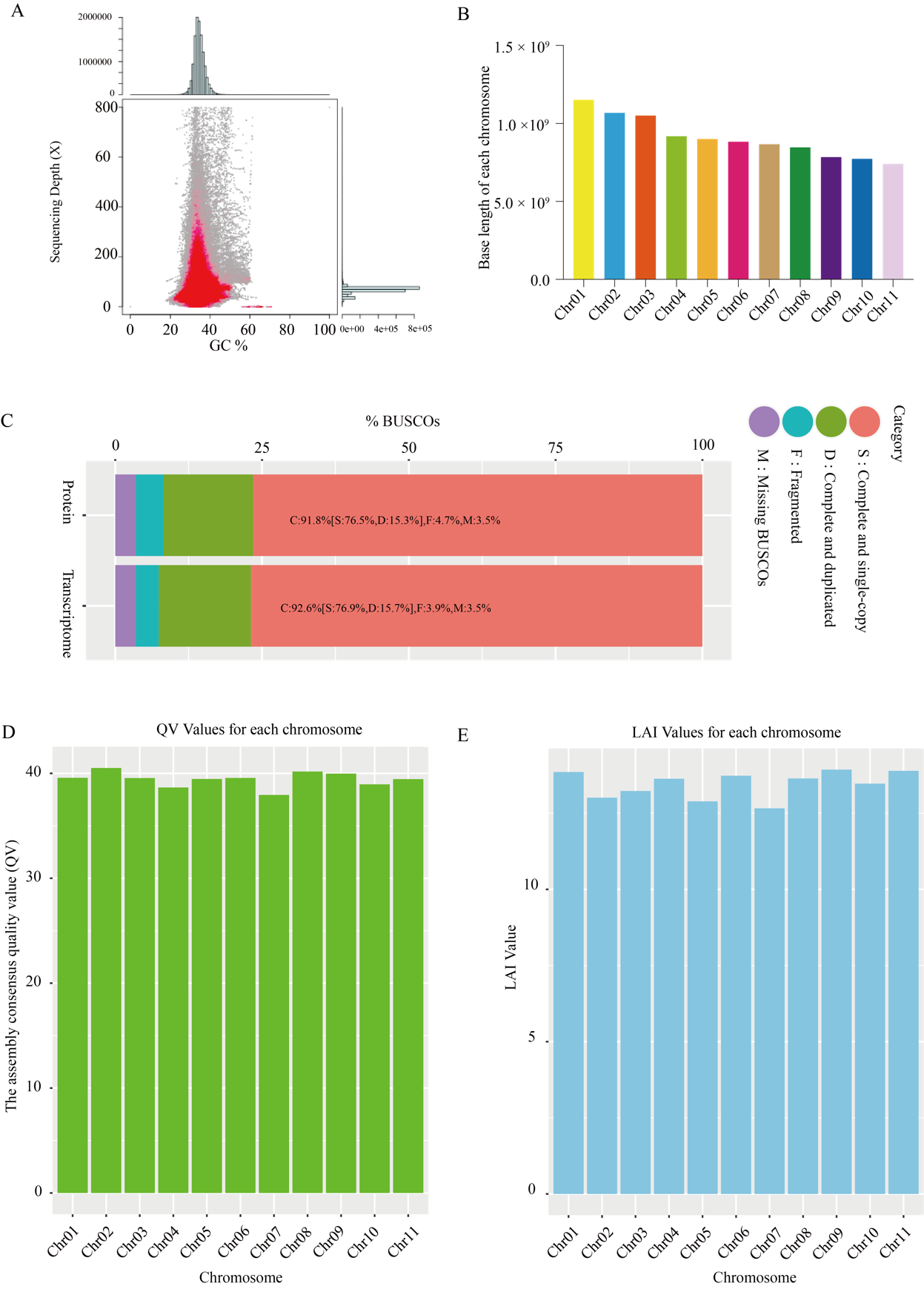
*

**Supplementary Figure S**2. Profile of genome assembly for *F. hodginsii* and evaluation of genome assembly quality. (A) The scatter plot presenting the GC content distribution under different sequencing depths based on long reads from the PacBio platform. (B) The bar plots presenting the base length for each assembled chromosome. (C) The bar plots presenting the BUSCO score. (D) The assembly consensus quality value representation for each assembled chromosome*.* (E) LAI value representation for each assembled chromosome*.*

*
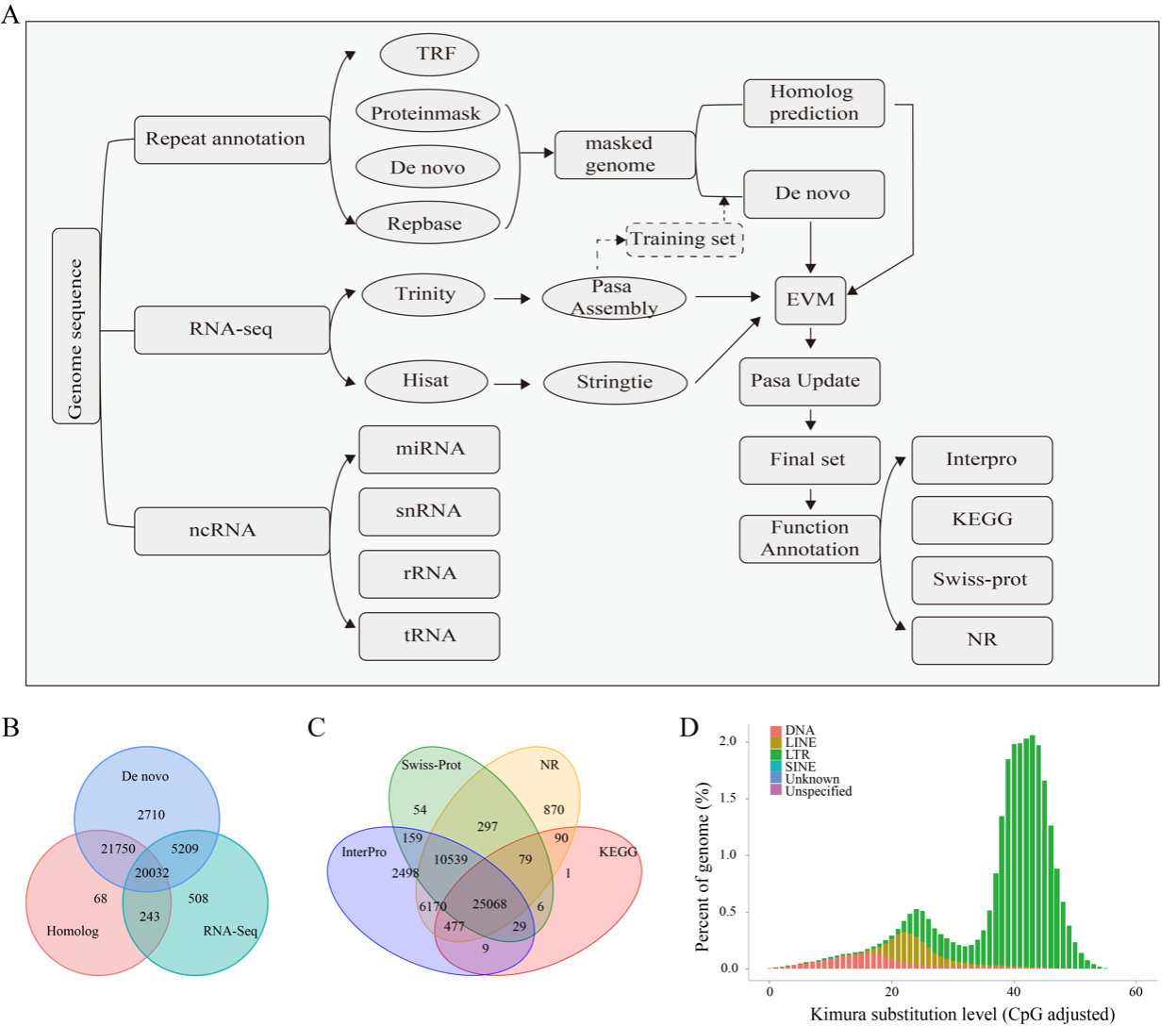
*

**Supplementary Figure S**3. Annotation of the *F. hodginsii* genome. (A) Gene annotation flowchart. (B) Venn diagram illustrating the annotated number using three different methods. (C) Venn diagram showing the overlapped genes with functional annotation by homology searches against different databases. (D) Bar plot graphs showing the percentage of TE under different Kimura substitution level.


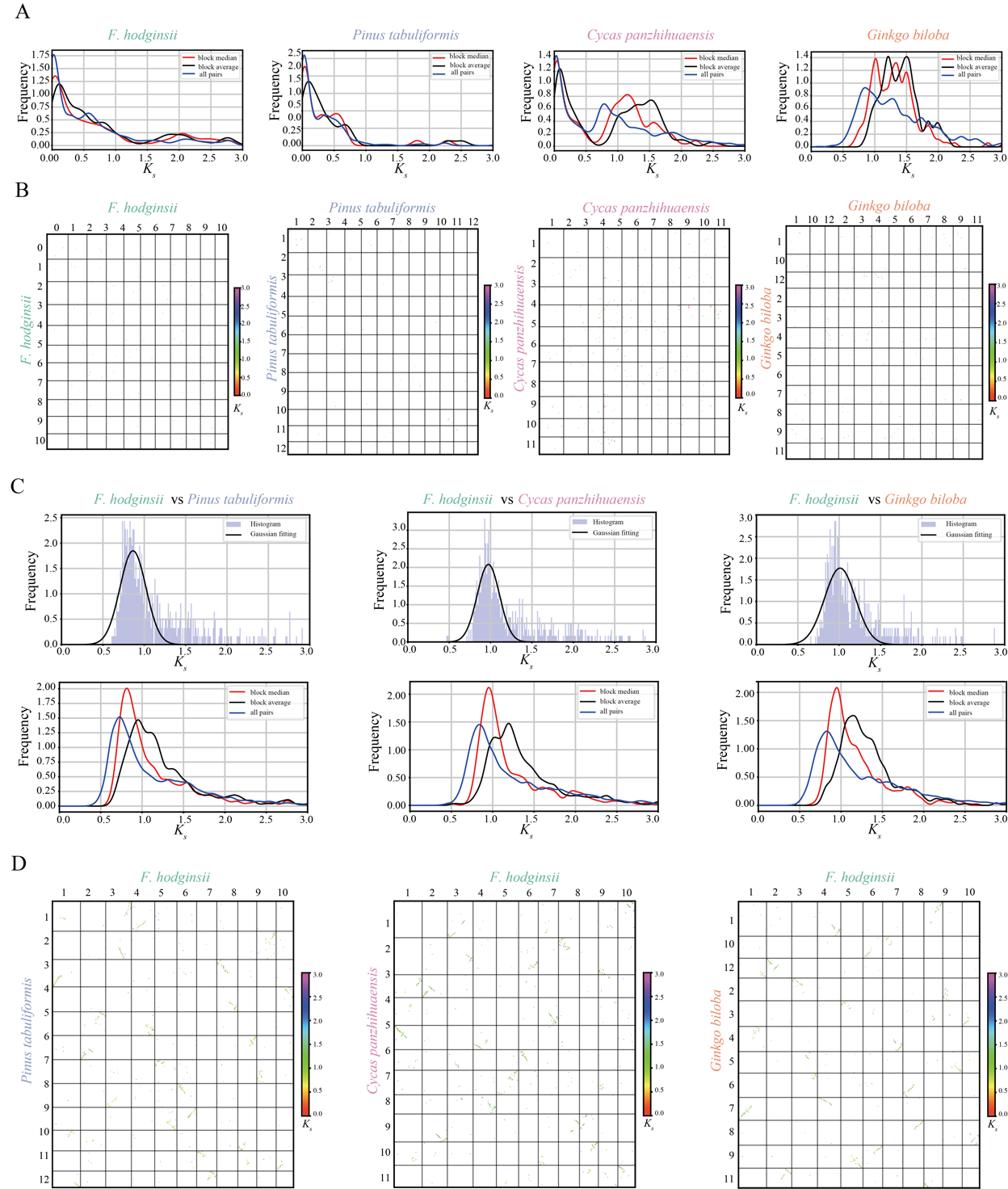


**Supplementary Figure S**4. Evolutionary analyses of whole-genome duplications. (A) Ks frequency of paralogs based on inter-genomic comparison in four gymnosperms. (B) Dotplot of syntenic gene pairs in four gymnosperms. (C) Ks frequency of orthologs between *F. hodginsii* and other gymnosperms. (D) Dotplot of orthologs between *F. hodginsii* and other gymnosperms


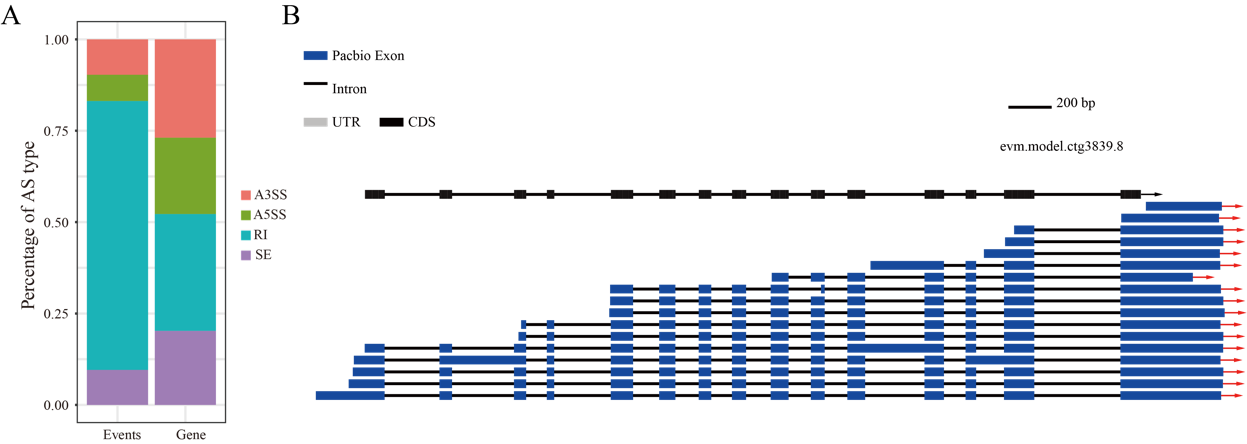


**Supplementary Figure S**5. Analysis of alternative splicing in *F. hodginsii*. (A) Histogram showing the percentage of AS events and genes. (B) Structure of PacBio Iso-seq reads presenting three intron retention events.


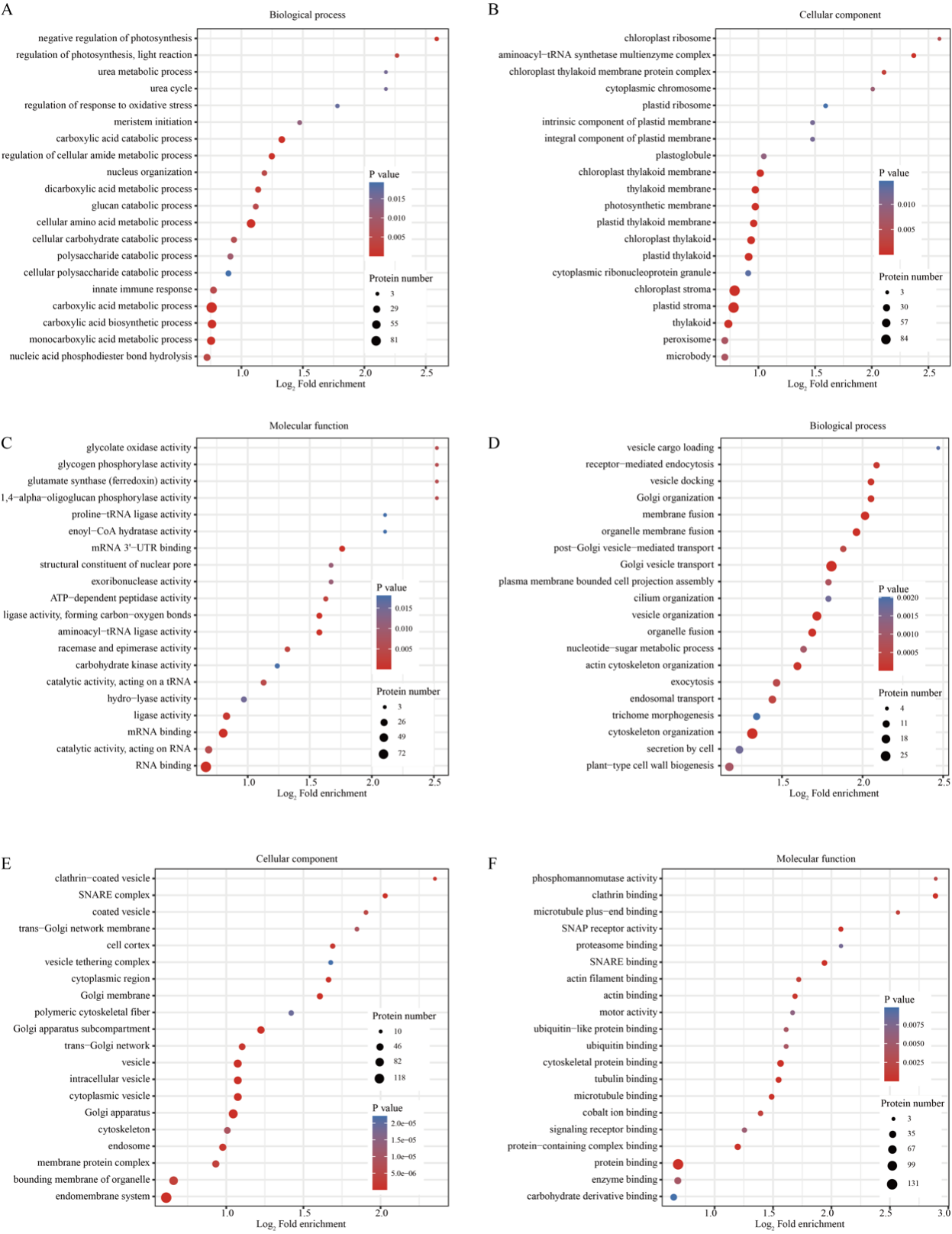


**Supplementary Figure S**6. Enriched GO term for differentially expressed proteins. Up-regulated proteins presented enriched GO terms in three categories: biological process (A), cellular component (B), and molecular function (C). Similarly, down-regulation proteins presented enriched GO terms in biological process (D), cellular component (E), and molecular function (F).


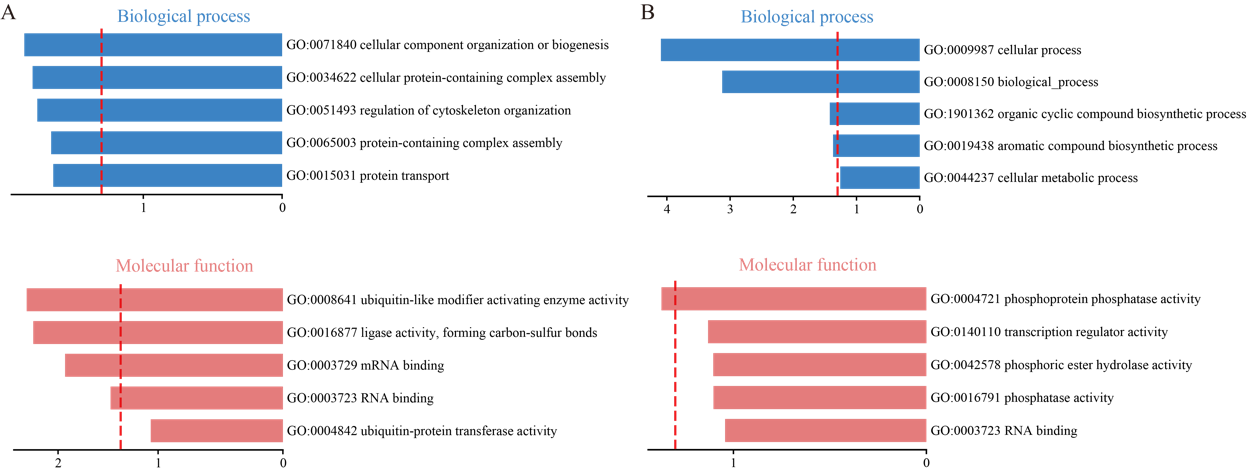


**Supplementary Figure S**7. Enriched GO term for genes with shorter (A) and longer 3’UTR (B).


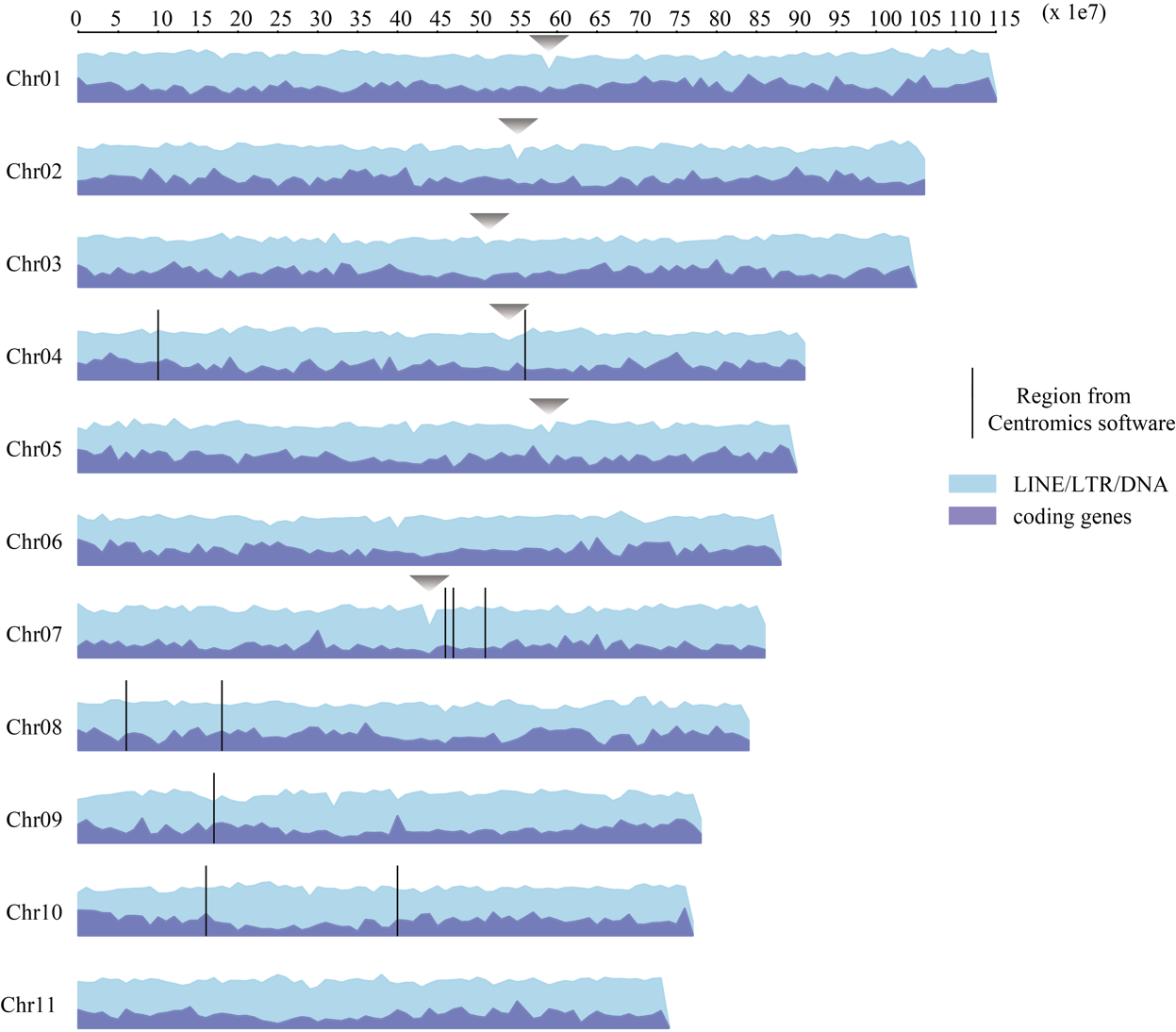


**Supplementary Figure S**8. Stack plots representation for the chromosome sizes, density of the gene, and repeat elements.
